# PDP-Miner: an AI/ML tool to detect prophage tail proteins with depolymerase domains across thousands of bacterial genomes

**DOI:** 10.1093/bioinformatics/btaf460

**Published:** 2025-08-21

**Authors:** Jeff Gauthier, Irena Kukavica-Ibrulj, Roger C Levesque

**Affiliations:** Institut de Biologie Intégrative et des Systèmes, Université Laval, Québec, QC, G1V 0A6, Canada; Département de Microbiologie, d’Infectiologie et d’Immunologie, Faculté de Médecine, Université Laval, Québec, QC, G1V 0A6, Canada; Institut de Biologie Intégrative et des Systèmes, Université Laval, Québec, QC, G1V 0A6, Canada; Département de Microbiologie, d’Infectiologie et d’Immunologie, Faculté de Médecine, Université Laval, Québec, QC, G1V 0A6, Canada; Institut de Biologie Intégrative et des Systèmes, Université Laval, Québec, QC, G1V 0A6, Canada; Département de Microbiologie, d’Infectiologie et d’Immunologie, Faculté de Médecine, Université Laval, Québec, QC, G1V 0A6, Canada

## Abstract

**Motivation:**

Antibiotic resistance is predicted to become the leading cause of human mortality by 2050. Despite this, no other major antibiotic class has been approved for medical use since 1987. Nevertheless, phage tail proteins offer a promising alternative, given their depolymerase activity toward outer membrane polysaccharides. Several pathogenic bacteria harbor prophages, thus making these prophages’ molecular target already known.

**Results:**

We therefore developed a wrapper for an existing machine learning-based phage depolymerase prediction tool (Depolymerase-Predictor), called PDP-Miner, which annotates phage tail proteins *ab initio*, detects depolymerase activity within this candidate protein subset, and then performs post-hoc validation by annotating protein domains thereby allowing the user to investigate for protein domains indicative of depolymerase activity. This tool allowed identification of 10 high confidence phage depolymerase gene candidates across all 1294 *Pseudomonas* genomes available on the International Pseudomonas Consortium Database while also accurately reporting depolymerases in known phage genomes, similarly to other software like PhageDPO or DepoScope.

**Availability and implementation:**

Source code, test datasets and documentation are freely available for download at http:///www.github.com/jeffgauthier/pdpminer. This software is free and open source under the GNU General Public License v3.0.

## 1 Background

Antibiotic resistance will become the leading cause of human mortality by 2050, surpassing cancer and cardiovascular disease (Antimicrobial Resistance Collaborators 2022). Despite this, no major antibiotic class has been approved for medical use since 1987 (Hutchings *et al.* 2019). There is an urgent need for alternative antibiotics classes to mitigate AMR. Bacteriophages have been used as antibacterial treatments as early as in 1919 (Barron 2022), even before the discovery of penicillin (Fleming 1929). Phage therapy remains popular in Eastern European countries (Yang *et al.* 2023). However, standardization issues and their popularity in twentieth century Soviet republics may have contributed to their early dismissal in Western medicine in favor of broad-spectrum compounds (Loc-Carrillo and Abedon 2011).

Perhaps the most important caveat with phages is their narrow host range, often down to the strain level (Fong *et al.* 2021). Indeed, bacteriophages adhere to the cell wall via tail proteins (Taslem Mourosi *et al.* 2022) via highly specific binding to either: outer membrane receptors, lipopolysaccharides (LPS), exopolysaccharides (EPS), and capsule polysaccharides (CPS) or a combination of those. Several tail proteins also exhibit depolymerase activity toward specific components (Knecht *et al.* 2019). Therefore, using phage depolymerases (PDPs) to weaken the bacterial cell wall constitutes a potential treatment strategy (alone or in combination with antibiotics), assuming the PDP’s target is well known. This strategy has already delivered promising results in >20 *in vivo* trials (Wang *et al.* 2024) (see Table 1, available as supplementary data at *Bioinformatics* online). However, most PDPs currently described were purified from lytic phages, which requires continuous culture to either maintain the phage or find its target.

Several pathogenic bacteria, e.g. *Pseudomonas aeruginosa*, are known to harbor prophages (up to 7 per genome) (Johnson *et al.* 2022), implying that any PDP encoded by these prophages would have its molecular target already known. PDPs can therefore be purified and administered as a bactericidal treatment to other strains having similar extracellular polysaccharide structure, all without having to cultivate the source phage and host. Alternative strategies to produce a prophage tail protein would be complete gene synthesis, plasmid cloning, and expression at high levels. This in turn reduces the likelihood of genetic alterations to either the PDP or its source organism. Prophage DNA can be annotated from the host bacterial genomic DNA sequence.

In this study, we have investigated the presence of PDPs across 1294 *P. aeruginosa* genomes from the International Pseudomonas Consortium Database (IPCD) (Freschi *et al.* 2015) (http://ipcd.ibis.ulaval.ca). Initially, the SVM-based tool Depolymerase-Predictor (DePP) was used alone because of this training set comprised experimentally verified phage tail depolymerases (Magill and Skvortsov 2023), but it yielded several false-positive results when all protein-coding genes were considered as input (Table 1, available as supplementary data at *Bioinformatics* online). We therefore developed a wrapper for this software, called PDP-Miner, which annotates tail proteins *ab initio* then runs DePP exclusively on this subset. PDP-Miner also performs post-hoc validation by annotating protein domains thereby allowing the user to investigate for protein domains indicative of glycosyl hydrolase activity, among others.

This discovery pipeline could help mitigating AMR in *P. aeruginosa*, which is a major widespread human opportunistic pathogen (Weimann *et al.* 2024) infecting patients with cystic fibrosis (CF), chronic obstructive pulmonary disease (COPD), and large burn wounds, all while causing other soft tissue and systemic infections (Morin *et al.* 2021). *Pseudomonas aeruginosa*, a WHO top priority pathogen regarding AMR emergence (WHO 2024), accounts for half a million annual deaths (Ikuta *et al.* 2022).

## 2 Software, data, and implementation

### 2.1 Type strain dataset (four genomes)

Genomic FASTA sequences from *P. aeruginosa* strains PAO1, PAK, LESB58 and *P. paraeruginosa* strain PA7 were retrieved from the Pseudomonas Genome Database (http://www.pseudomonas.com) to form a small dataset of phenotypically and genomically well-described strains in which PDP gene candidates are expected to be discovered. Indeed, PDP activity has been experimentally verified against these four type *Pseudomonas* strains in PAO1 (Olszak *et al.* 2017), PAK (Bhattacharjee *et al.* 2022), as well as the presence of phage-tail-like bacteriocins in PA7 (Saha *et al.* 2023) and recently acquired prophage islands in Liverpool Epidemic Strain LESB58 (Winstanley *et al.* 2009).

### 2.2 IPCD *Pseudomonas* genome dataset

All 1294 *Pseudomonas* genomes available on the IPCD (Freschi *et al.* 2015) were selected and run through PDP-Miner. Beyond archiving source isolates, this database also routinely generated Illumina MiSeq 2x300 bp genome data assembled with the A5-miseq pipeline (Coil *et al.* 2015) for all isolates. Therefore, any potential PDP candidates found via PDP-Miner could be tested against the original strains and/or strains having a similar surface polysaccharide composition.

### 2.3 PDP-Miner algorithm

**Figure 1.**
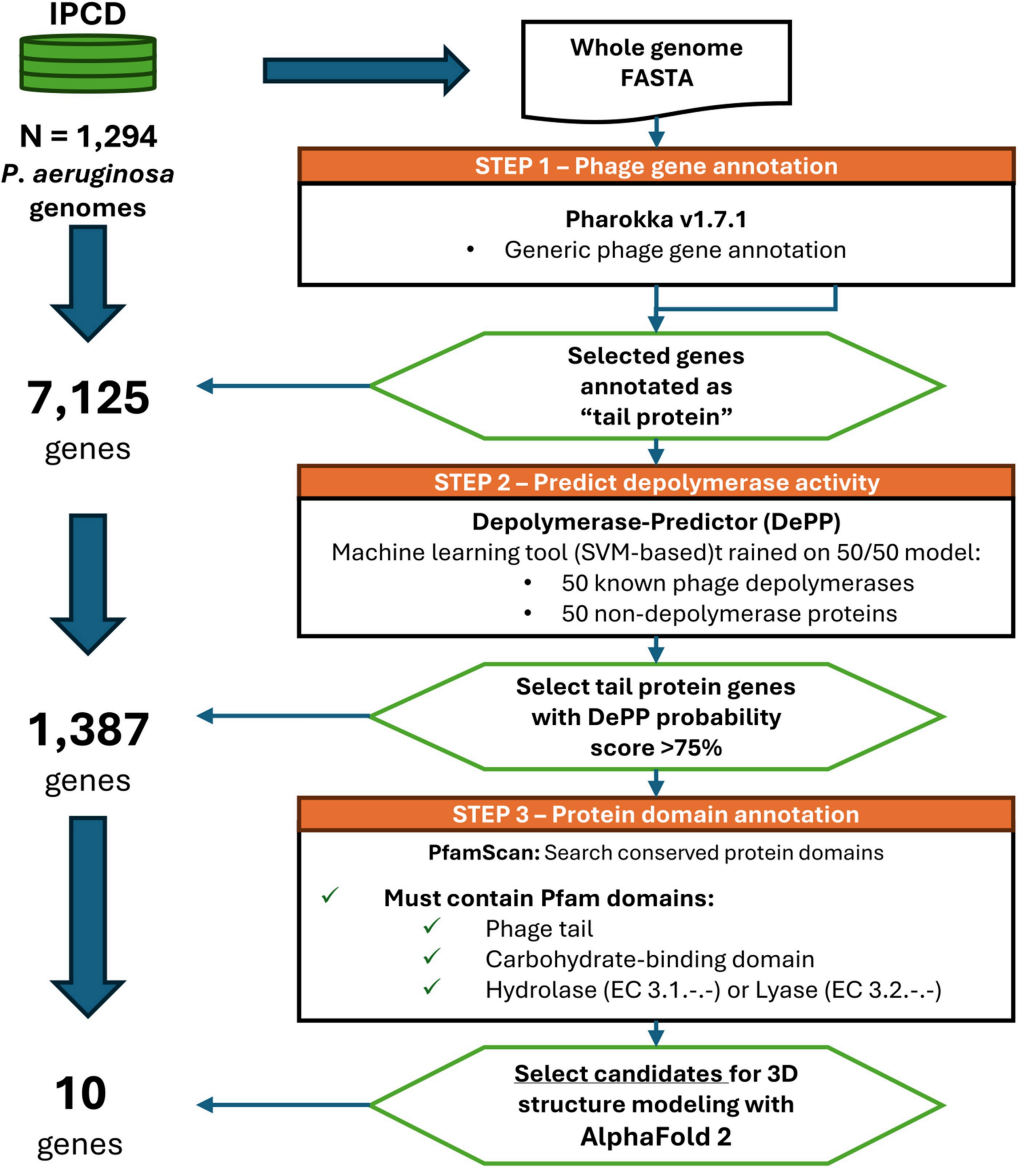
Flowchart describing the PDP-Miner pipeline. Briefly, prophage genes are annotated in silico with Pharokka (Bouras *et al.* 2023). Then, tail protein genes are scanned with Depolymerase-Predictor (DePP, Magill and Skvortsov 2023) to obtain a probability score associated with each annotation. Finally, tail spike protein genes with >75% DePP probability are investigated for potential domains indicative of glycosyl-hydrolase/lyase activity with PfamScan. Those candidate tail protein depolymerase genes are then selected for homology modeling with AlphaFold 2.

An overview of the PDP-Miner algorithm is provided in Fig. 1.

Briefly, genome FASTA sequences are annotated with Pharokka v1.7.4 (Bouras *et al.* 2023) to list only phage-related genes. Then, phage tail protein genes are selected with seqtk v1.4 (https://github.com/lh3/seqtk) from a list of loci that were labeled “tail protein” by Pharokka. Then, Depolymerase-Predictor v1.0.0 (DePP, Magill and Skvortsov 2023), a SVM-based phage depolymerase classifier, assigns a probability score to only phage tail protein genes (i.e. their amino acid translation). DePP returns a 2-column table with gene IDs and probability scores. Pharokka and DePP annotations are then merged into a single table and only gene candidates with a DePP probability over 75% are kept. To further assess the validity of DePP predictions, PfamScan v1.6 (Mistry *et al.* 2021) is run on candidate gene products with over 75% DePP score. Domains found by PfamScan are inserted into the Pharokka/DePP table to allow the user to search for domains indicative of depolymerase activity, e.g. hydrolase (EC 3.1.-.-), lyase (EC 3.2.-.-), carbohydrate binding domains, phage tail domains, and more. This allows the user to judge and prioritize annotations by validating DePP’s prediction, which is known by us to generate false positives when non-phage genes are considered (Table 1, available as supplementary data at *Bioinformatics* online).

### 2.4 Code and data availability

All IPCD genome sequences used in this study are publicly available through the NCBI BioProject primary accession code PRJNA325248. Raw sequencing data and strain metadata are available upon request at http://ipcd.ibis.ulaval.ca. The current implementation of PDP-Miner is publicly available on GitHub in the repository: http://www.github.com/jeffgauthier/pdpminer.

## 3 Benchmarking

### 3.1 Initial type strain dataset (four genomes)

The initial stage of the PDP-Miner workflow is to annotate phage tail proteins with Pharokka and evaluate their likelihood of being a depolymerase with Depolymerase-Predictor (DePP). This was initially tested with a restricted set of four *Pseudomonas spp*. type strain genomes (LESB58, PAK, PAO1 and *P. paraeruginosa* PA7). Among those, five putative phage tail proteins annotated by Pharokka were found with a DePP score above 75% (Fig. 1, available as supplementary data at *Bioinformatics* online). More precisely, two candidates were found for PA7 and one was found for each other strain. For these five candidate PDPs, the DePP score, i.e. the likelihood of them having depolymerase activity, ranged between 86% and 94%. In addition, we found by multiple sequence alignment that the nearest protein among DePP’s experimentally verified protein training set was the tail fiber protein of Klebsiella phage GH-K3 (Fig. 2, available as supplementary data at *Bioinformatics* online), suggesting high structural similarity between the top five and GH-K3’s tail fiber protein.

However, DePP by itself only reports a two-column table with sequence IDs and probability scores, which limits the interpretation of this dataset. To further help interpret the results, PDP-Miner uses PfamScan to annotate protein domains and report them alongside DePP score. Interestingly, phage-tail-3 and DUF1983 domains were found in GH-K3’s tail fiber protein and in each of the top five candidates, except the one in LESB58 which had only a phage-tail-3 domain (Fig. 3, available as supplementary data at *Bioinformatics* online). One protein, CDS #2309 from PA7, also had a “CBM_5_12” domain (Pfam domain 02839), which is a “carbohydrate binding domain found in many glycosyl hydrolase enzymes” (NCBI 2024).

To further verify structural similarity between GH-K3 and PA7 CDS #2309, and thereby its likelihood of being a phage tail depolymerase, we predicted their 3D structure with AlphaFold 2 via the NeuroSnap webserver (https://neurosnap.ai). The 3D structure of Depo32 was used for guiding predictions as this structure was obtained through cryogenic electron microscopy (see PDB structure #7VYV). *P. paraeruginosa* PA7 CDS #2309 and GH-K3’s tail fiber protein had highly similar overlapping structures (Fig. 4, available as supplementary data at *Bioinformatics* online).

### 3.2 IPCD *Pseudomonas* genome dataset

Among the 1271 *Pseudomonas* genomes investigated in this study, 1387 phage tail proteins, stemming from 735 isolates, had a DePP score above 75%. However, 784 of those had no annotated Pfam domain and 496 of them had only a “Phage-tail_3” domain annotated. Nevertheless, among the remaining 107 phage tail proteins, ten harbored domains indicative of depolymerase activity (Table 2, available as supplementary data at *Bioinformatics* online).

More precisely, eight had both a LysM motif (PF01476) and a transglycosylase SLT domain (PF01464) while two others had a PA2794-like exo-alpha-sialidase C-terminal domain (PF22432). These ten candidate PDPs, while not having the highest DePP score, have evidence that they contain domains capable of degrading cell wall polysaccharides and/or peptidoglycans. This implies an *a priori* discovery rate of one candidate PDP per 100 isolates among the IPCD collection, strictly via genomic data mining.

### 3.3 Cross-comparison against known phages

To evaluate PDP-Miner’s reliability with respect to other similar software, a comparative analysis was made by launching PDP-Miner (this study), PhageDPO (Vieira *et al.* 2025) and DepoScope (Concha-Eloko *et al.* 2024) against five phage known phage genomes: *Klebsiella* phage GH-K3 (NC_048162.1); *Acinetobacter* phage IME200 (NC_028987.2); *Klebsiella* phage KP32 (NC_013647.1); *Klebsiella* phage KP36 (NC_029099.1) and *Klebsiella* phage NTUH-K2044-K1-1 (NC_025418.1), all of which harbor experimentally verified depolymerase genes (Lin *et al.* 2014, Majkowska-Skrobek *et al.* 2016, Liu *et al.* 2019, Squeglia *et al.* 2020, Cai *et al.* 2023).

We selected phage genomes instead of bacterial genomes with prophages, to accommodate input requirements for PhageDPO and DepoScope, both of which were designed to annotate sequences from phage genomes. To avoid gene annotation bias between tools, multi-FASTAs containing genes annotated as “tail protein” by Pharokka were used as the starting input for all tools (except PDP-Miner, which generates this data as part of its workflow).

Comparative results are reported in Table 3, available as supplementary data at *Bioinformatics* online, along with gene coordinates and Pfam domain hits. At least one tail protein per phage was consistently called by all tools with >90% prediction score, except for phage GH-K3 (Table 3, available as supplementary data at *Bioinformatics* online). Interestingly, no Pfam domain hits were found for any of these four high scoring genes despite their high prediction scores.

Furthermore, the highest scoring gene for phage GH-K3 with PDP-Miner, CDS 0034, had Pfam domain NLPC_P60 (PFAM: PF00877), which is characteristic of NlpC/P60 peptidoglycan hydrolases (Griffin *et al.* 2023). Both PhageDPO and DepoScope scored this gene with a 0% value, despite the presence of a domain suggesting a potential cell wall degrading activity (Table 3, available as supplementary data at *Bioinformatics* online). Another gene bearing the same domain, KP36_CDS_0048, was the second best scored gene by PDP-Miner for phage KP36 despite 0% scores by PhageDPO and DepoScope.

Both these sequences were aligned against a set of eight *Klebsiella* phage depolymerases with MUSCLE v3.8.3.1551 (Edgar 2004) and clustered in a neighbor-joining tree with Jalview v2.11.4.1 (Waterhouse *et al.* 2009). The amino acid sequences of GH-K3_CDS_0034 and KP36_CDS_0048 formed a monophyletic cluster in a neighbor-joining tree (Fig. 5, available as supplementary data at *Bioinformatics* online) with KP32gp38 (PDB: 6TKU), a capsule polysaccharide (CPS) depolymerase with CPS serotype K21 specificity (Majkowska-Skrobek *et al.* 2018). Interestingly, the only candidate PDB that was consistently found by all three tools in KP32 (CDS_0038) shares little homology to KP32gp38, despite what its CDS tag would suggest (Fig. 6, available as supplementary data at *Bioinformatics* online).

## 4 Significance

We developed a wrapper for Depolymerase-Predictor, called PDP-Miner, which annotates tail proteins *ab initio*, and then runs DePP exclusively on this subset while listing Pfam domains to further allow researchers to validate predictions made by DePP. This software was used to investigate the presence of prophage-borne depolymerases across 1294 *P. aeruginosa* genomes from the IPCD. PDP-Miner successfully recovered gene candidates whose amino acid translation had similar predicted 3D structures to known phage depolymerase structures. The contextualization of prediction scores with protein domain annotations also helped re-assess candidate genes with ambiguous prediction scores, even when a putative depolymerase domain was found.

To our knowledge, this is the first implementation of a tool that predicts phage depolymerase genes within several whole prokaryotic genomes, more precisely within prophages. If the infected host’s serotype is known, then this allows to predict a candidate depolymerase’s specificity for conspecific strains. In addition, as evidenced by the cross-comparison mentioned above, PDP-Miner can be run on phage genomes as well.

## 5 Known limitations

One limitation of this approach is whether the prophages have been rendered defective through neutral evolutionary forces over time, e.g. mutation and genetic drift (Bobay *et al.* 2014). In this case, tail protein genes could drift due to the absence of selective pressure for resuming a lytic cycle. In any case, verifying a candidate depolymerase’s activity and specificity would require expressing and purifying the protein and then assessing its lytic ability *in vitro*.

PDP-Miner is also dependent on DePP’s intrinsic accuracy to predict depolymerase activity. As mentioned above, DePP outputs high probability scores even to non-phage proteins, suggesting that its learning model makes it recognize domains interacting with polysaccharides regardless of them being phage-associated or not (Table 1, available as supplementary data at *Bioinformatics* online). Among the IPCD genomes investigated, there were some instances of genes having high DePP scores (>90%) but without glycosyl hydrolase domains found. This could be attributable to stringent parameters passed to PfamScan, or to domains that have not yet been characterized. One example of this was the DUF1983 domain found in four of five of the type strains’ top scoring gene candidate. DUF stands for “Domain of Unknown Function” in Pfam nomenclature, however its association with both Klebsiella phage GH-K3 tail fiber protein and those top scoring candidates was notable, given the strong homology between all those (Fig. 3, available as supplementary data at *Bioinformatics* online). Also, PDP-Miner relies on Pharokka (Bouras *et al.* 2023), itself a wrapper for PHANOTATE (McNair *et al.* 2019), to refine the input space to tail proteins only, though there are other software such as PhageTailFinder (Zhou *et al.* 2023) that could achieve the same purpose.

As all machine learning tools, regardless of learning strategy, the quality of training data is paramount to the tool’s accuracy and recall capabilities (Gupta *et al.* 2021). DePP was trained on a 50/50 model including 50 experimentally verified phage depolymerase proteins and a random set of 50 proteins unrelated to phages (Magill and Skvortsov 2023). This dataset does not cover the diversity of phage depolymerases both from a taxonomic and structural point of view. Nevertheless, DePP allows for custom models to be built, which could be done when more experimental data will be available.

## Supplementary Material

btaf460_Supplementary_Data

## Data Availability

Source code, test datasets and documentation are freely available for download at http:///www.github.com/jeffgauthier/ pdpminer. This software is free and open source under the GNU General Public License v3.0.
