# Supplementary material for: PDP-Miner: an AI/ML tool to detect prophage tail proteins with depolymerase domains across thousands of bacterial genomes
Source: Bioinformatics. 2025 Aug 21;41(11):btaf460. doi: 10.1093/bioinformatics/btaf460 (PMC12579547; doi:10.1093/bioinformatics/btaf460)
Supplement: btaf460_Supplementary_Data [file btaf460_supplementary_data.docx]

**PDP-Miner: an AI/ML tool to detect prophage tail proteins with depolymerase domains across thousands of bacterial genomes**

Jeff Gauthier^1,2*^, Irena Kukavica-Ibrulj^1,2^, Roger C. Levesque^1,2,*^

1. Institut de Biologie Intégrative et des Systèmes, Université Laval, Québec, QC, Canada
2. Département de microbiologie, d’infectiologie et d’immunologie, Faculté de médecine, Université Laval, Québec, QC, Canada

* Corresponding authors : [jeff.gauthier.1@ulaval.ca](mailto:jeff.gauthier.1@ulaval.ca), [rclevesq@ibis.ulaval.ca](mailto:rclevesq@ibis.ulaval.ca)

# Supplementary Figures


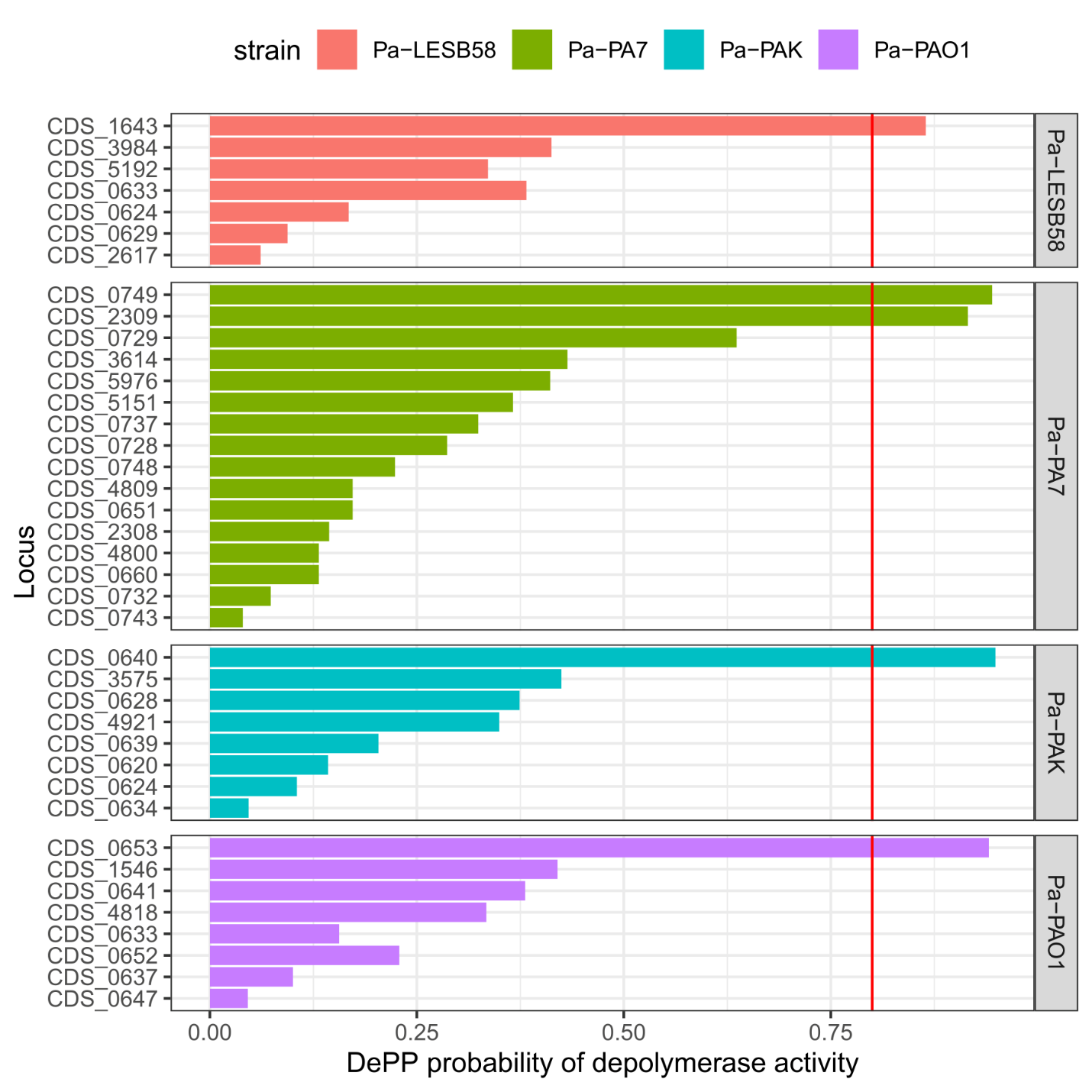


**Suppl. Figure 1.** **Phage tail proteins among four type *Pseudomonas aeruginosa* genomes PAO1, LESB58, PAK and PA7, sorted by DePP score.** CDS: coding DNA sequence. DePP: Depolymerase-Predictor. The red vertical length indicates the user-specified threshold for selecting candidate phage depolymerases deserving further characterization at the domain level.


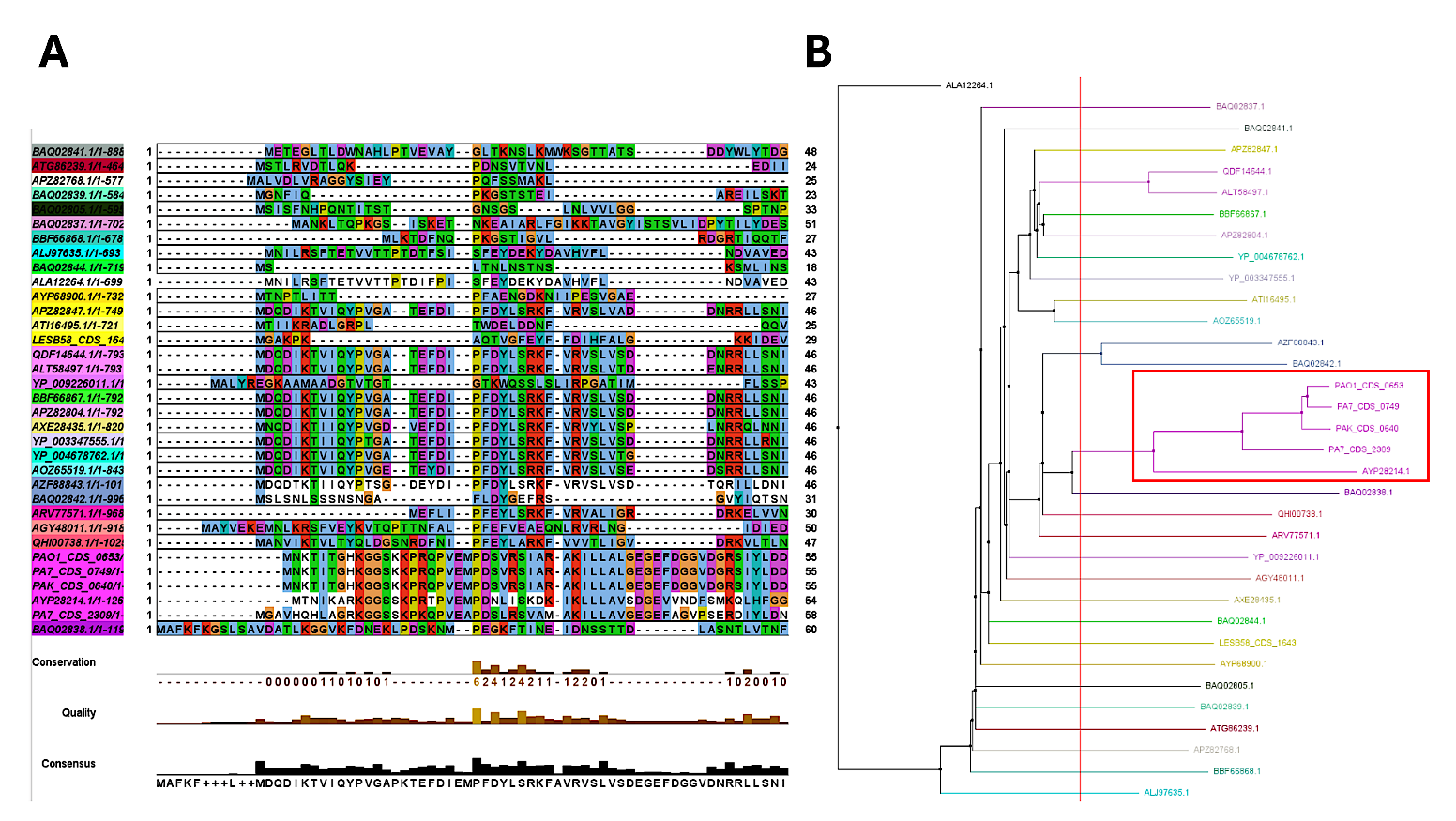


**Suppl. Figure 2.** **GH-K3 phage tail fiber protein (AYP28214.1) is the nearest neighbor to the top 5 PDPs found by PDP-Miner.**  A : Multiple sequence alignment of the top 5 PDPs found in four *Pseudomonas* type strains, and DePP sequences used for model training. B: Neighbor-joining tree calculated from the alignment in A.


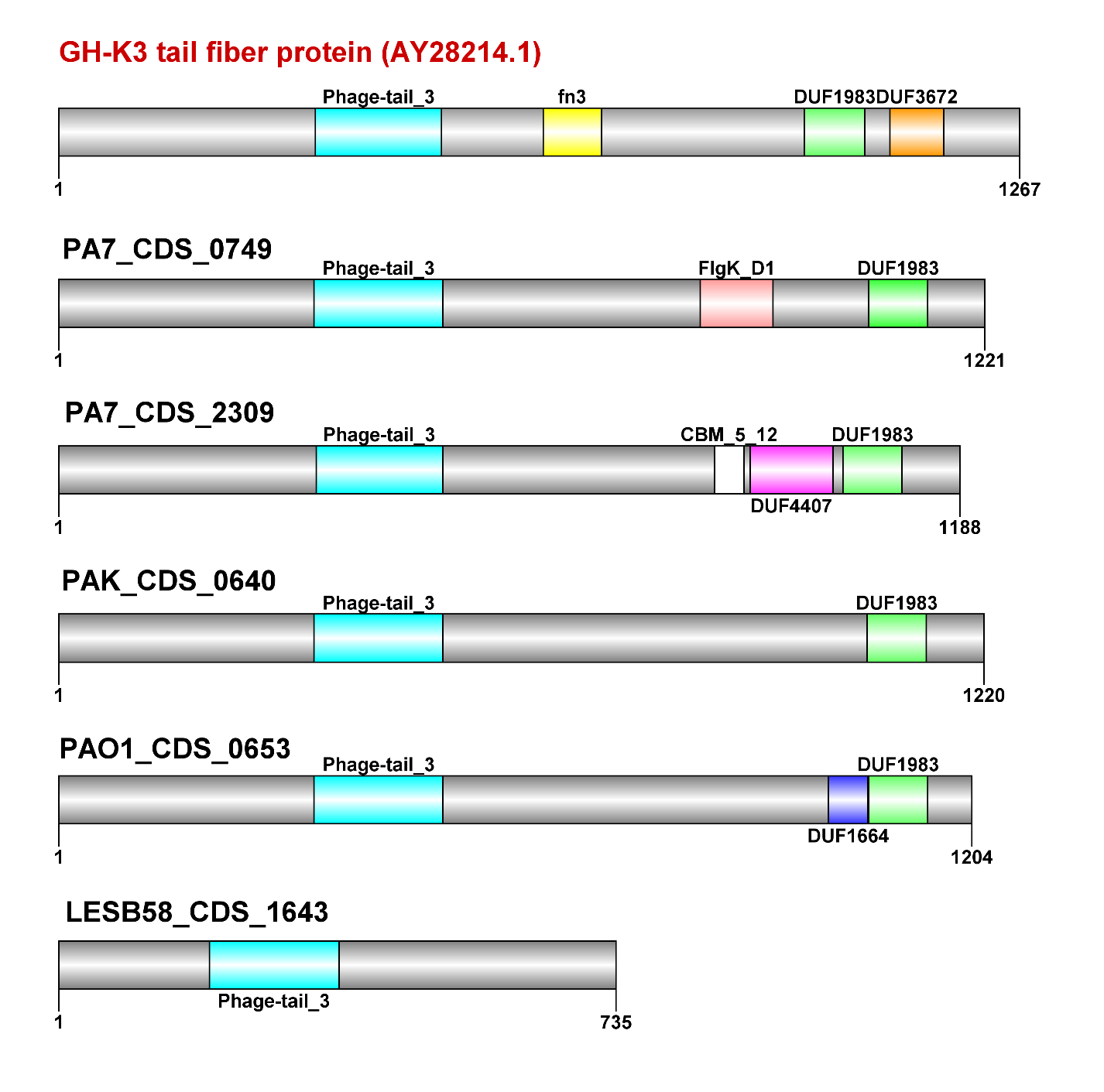


**Suppl. Figure 3.** **Protein domains of top scoring proteins among type *Pseudomonas aeruginosa* and *paraeruginosa* strains.** All domain annotations follow Pfam nomenclature (fn3: fibronectin III, CBM: carbohydrate binding domain, flgK: flagellin K, DUF: domain of unknown function).


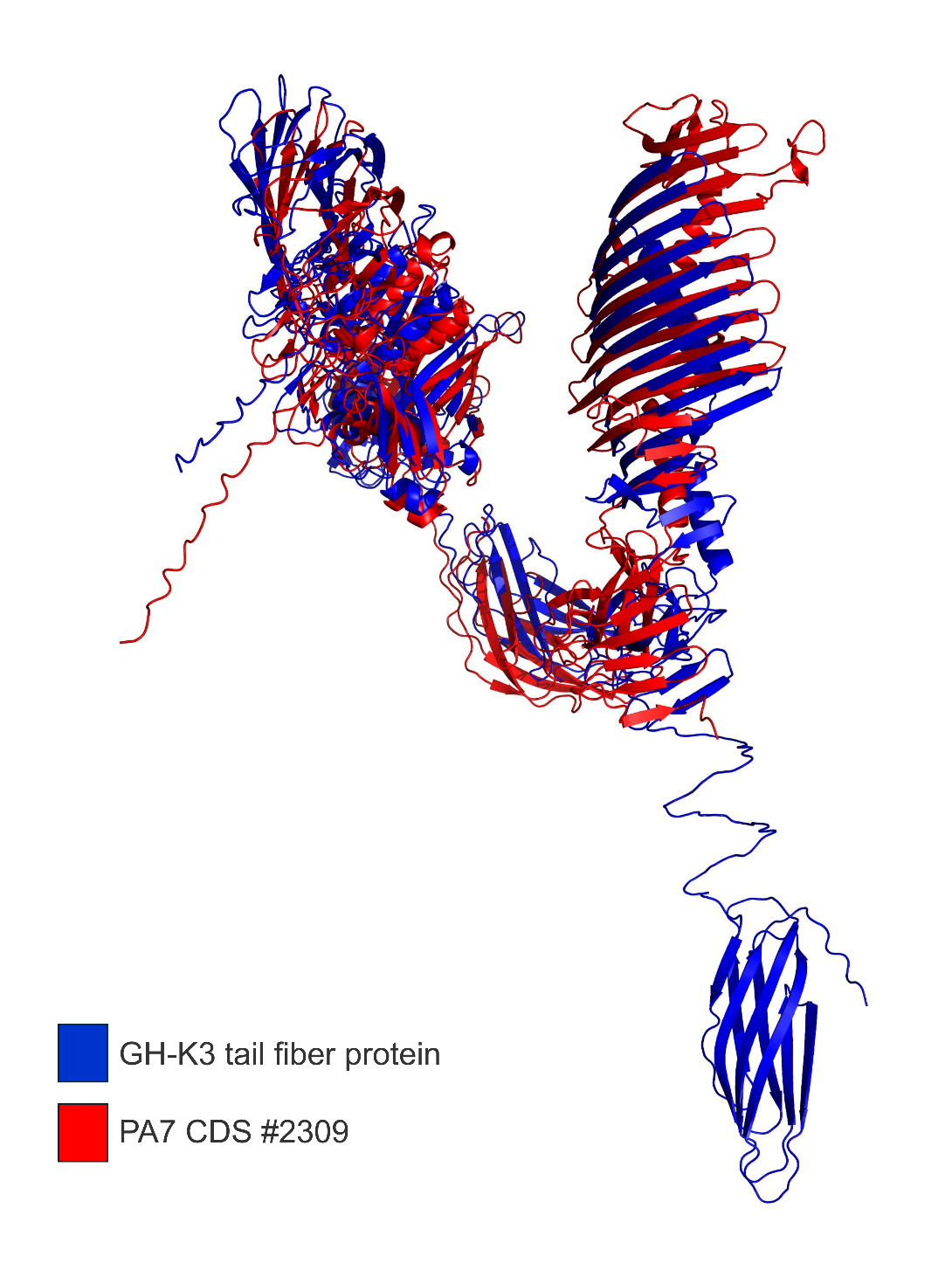


**Suppl. Figure 4.** **Comparing the predicted structures of Klebsiella phage GH-K3 tail fiber protein (blue) and *P. paraeruginosa* PA7 CDS # 2309 (red).** Note that the monomer form of Klebsiella phage depolymerase Depo32 (PDB #7VYV) was used as a reference to guide both structure predictions; this protein natively forms a homotrimer. Protein structure visualizations were done with PyMOL3.


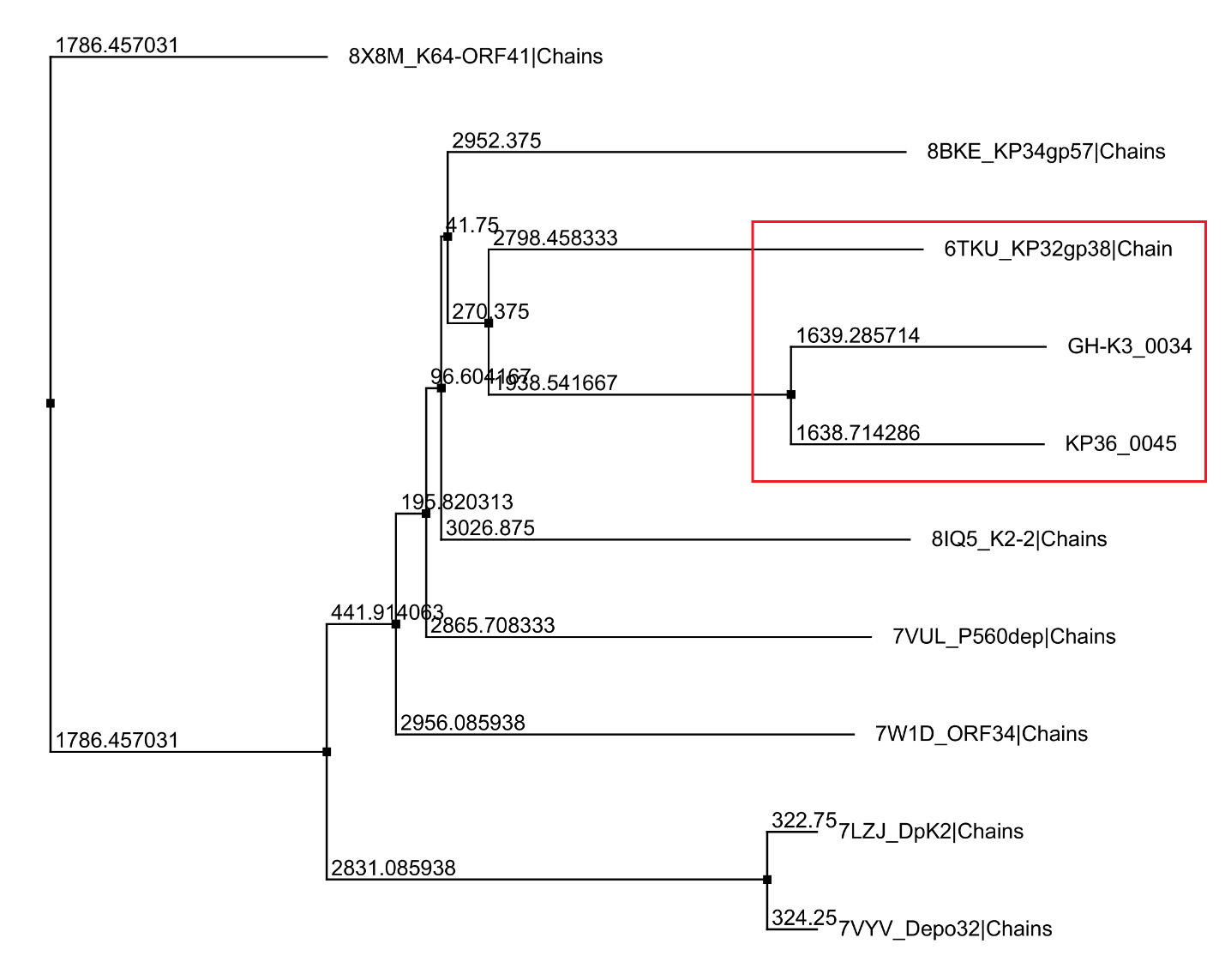


**Suppl. Figure 5.** Neighbor-joining tree of two minor tail proteins with a NlpC/P60 hydrolase domain despite a 38% prediction score for PDP-Miner and 0% for both PhageDPO and DepoScope.

MUSCLE (3.8) multiple sequence alignment

6TKU_KP32gp38|Chain ---MLDNFNQPKGSTIGVLKDGRTIQEAFDSLPRLESFSGSTATDKLRAAITL-----GV

KP32_CDS_0038 MALVSQSIKNLKG---GISQQPEILRYPEQGTLQVNGWSSETEGLQKRPPMVFIKSLGGR

: :.::: ** *: :: :. . :. .::.:*..* : *..:.: *

6TKU_KP32gp38|Chain SEVAIGP----------------VEGNGGRPYEF--------GD---VVIPYP---LRIV

KP32_CDS_0038 GYLGEDPYIHLINRDEYEQYYAVFTGNNVRVFDLSGYEYQVRGDRSYVTVNNPKDNLRMV

. :. .* . **. * ::: ** *.: * **:*

6TKU_KP32gp38|Chain GCGSQGINVTKGTVLKRSAGASFMFHFTGEGQAQRPMGGGLF----NINLNG--------

KP32_CDS_0038 TVADYTFIVNRTRQVRESQNLTNGGTFRDNVDALINVRGGQYGRKLEVNINGVWVSHQLP

.. : *.. :. * . : * .: :* : ** : ::*:**

6TKU_KP32gp38|Chain ---------------DTATALGDIIKVTQ--WSYFKAN---NC---------AFQNMAGW

KP32_CDS_0038 PGDNAKDDPPKVDAQAIAEAIAVLLRTAHPTWTFNVGTGFIHCIAPAGTTIDILETKDGY

* *:. ::..:: *:: .. :* ::. *:

6TKU_KP32gp38|Chain GIRLKDVMESNI-----------SGNLFRRLGGPSGGG----ILFDDVRSAVTDNVN-NL

KP32_CDS_0038 ADQLINPVTHYVQSFSKLPLNAPDGYMVKIVGDTSKTADQYYVKYDKSQKVWKETVGWNI

. .* : : : .* :.. :*..* . : :*. ... .:.*. *:

6TKU_KP32gp38|Chain HI--------------EDNTFALMSGPWIGSTA-----NSNPDL-------IWIVRNKFE

KP32_CDS_0038 SIGLDYTTMPWTLVRAADGNFDLGYHDWKDRRAGDEDTNPQPSFVNSTITDVFFFRNRLG

* *..* * * . * *.:*.: :::.**.:

6TKU_KP32gp38|Chain FDGTPAAPNTVDSYVLDFQQLSRAFIQDNGFTHFTTERNRYVGVLRVGATAVGTIKFEDN

KP32_CDS_0038 FISGENIVMSRTSKYFEFYPPSVANYTDDDPLDVAVSHNR-VSVLKY------AVSFAEE

* . : * ::* * * *:. .:...** *.**. ::.* ::

6TKU_KP32gp38|Chain LLFACESAGLI--AGGIVVSRG---NVNNQGSATTAIKQ--------FTNTSSKLCKLER

KP32_CDS_0038 LLLWSDEAQFVLSANGVLSAKTAQLDLTTQFDVSDRARPYGIGRNIYYASPRSSFTSIMR

**: .:.* :: *.*:: :. ::..* ..: . ::.. *.: .: *

6TKU_KP32gp38|Chain VINVQSNGNVSVGQQI-------LPDGYINMAELPGNTRLPSEYDADGETT----SVLRV

KP32_CDS_0038 YYAVQDVSSVKNAEDMTAHVPNYIPNGVYSING-SGTENFACVLTKGAPSKVFIYKFLYM

**. ..*. .::: :*:* .: .*. .:.. .. :. ..* :

6TKU_KP32gp38|Chain PANTQVRQWSVPKMYKDGLTVTKVTVRAKGAAAGAILSLQSGSTVLSTKSIDAG------

KP32_CDS_0038 DENIRQQSWSHWD-FGDG-----VEVMAANCINSTMYMLMRNAYNVWIAAVDFKKNSTDF

* . ..** . : ** * * * .. .:: * .: : ::*

6TKU_KP32gp38|Chain VWKNYVFYVKANQ---LQET---LQLRNT--GTADVLADGMVFGKVDYIDWDFAIAPGTL

KP32_CDS_0038 PFEPYRFHVDAKRSYHISETAYDIETNQTVVNVKDIYGASFSKGTVAICDSDGKITTYEP

:: * *:*.*:. :.** :: .:* .. *: . .: *.* * * *:.

6TKU_KP32gp38|Chain AAGAKYTTPN-QSYLDVAGMRVQAVSIPMF--------------DGPTTG--------LQ

KP32_CDS_0038 MGDSWNSTPDIRISGDIAGKDIVIGFLYDFQYVFSRFLIKQEQNDGTTSTEDAGRLQLRR

..:. :**: . *:** : : * **.*: .

6TKU_KP32gp38|Chain VWVEATSANGSFVVVMKN--------------DTGSELVTTVTRCRVRAFVSKGHHHHHH

KP32_CDS_0038 AWVNYQDT-GAFTVSVENGNREFNYLVNARVGSTGLRLGQKATTTGQYRFPVTGNALYQK

.**: .: *:*.* ::* .** * ..* * .*: :::

6TKU_KP32gp38|Chain -----------------------------

KP32_CDS_0038 VSLSSFNASPVSIIGCGWEGNYMRRANGI

**Suppl. Figure 6.** Pairwise alignment (CLUSTAL format) of KP32gp38 (retrieved from PDB entry 6TKU) and the only predictive hit in *Klebsiella* phage KP32 for PDP-Miner, PhageDPO and DepoScope.

# Supplementary Tables

**Suppl. Table 1.** Top scoring genes according to Depolymerase-Predictor (DePP) when a whole-genome annotation scheme (Prokka) is used instead of a phage gene annotator (Pharokka).

All genes with a DePP probability score below 80% were discarded. Interestingly, all top scoring gene candidates were not phage-related, but were rather chromosome-encoded phospholipases, endonucleases and notably the autotransporter esterase EstA involved in rhamnolipid production. This finding implies that DePP is well trained at finding hydrolases (EC 3.1.x.x) with carbohydrate and/or lipid binding domains, and not necessarily phage tail proteins with depolymerase activity.

| **Genome** | **Locus** | **Abbr.** | **Description** | **EC number** | **COG number** | **Size (bp)** | **DePP probability** |
| --- | --- | --- | --- | --- | --- | --- | --- |
| Pa-LESB58 | CDS_05654 | estA | Esterase EstA | 3.1.1.1 | COG3240 | 1,941 | 91% |
|  | CDS_02946 | rhsC_3 | Putative deoxyribonuclease RhsC | 3.1.-.- | COG3209 | 2,637 | 89% |
|  | CDS_01756 | rhsC_1 | Putative deoxyribonuclease RhsC | 3.1.-.- | COG3209 | 2,658 | 88% |
|  | CDS_04611 | plcN_2 | Non-hemolytic phospholipase C | 3.1.4.3 | COG3511 | 2,193 | 86% |
|  | CDS_00042 | cdiA_1 | 16S rRNA endonuclease CdiA | 3.1.-.- | COG3210 | 10,611 | 85% |
|  | CDS_02943 | cdiA_2 | 16S rRNA endonuclease CdiA | 3.1.-.- | COG3210 | 16,938 | 82% |
|  | CDS_01806 | plcN_1 | Non-hemolytic phospholipase C | 3.1.4.3 | COG3511 | 2,079 | 82% |
| Pa-PA7 | CDS_05594 | estA | Esterase EstA | 3.1.1.1 | COG3240 | 1,941 | 91% |
|  | CDS_02669 | cdiA_4 | DNase CdiA | 3.1.-.- | NA | 2,871 | 86% |
|  | CDS_04466 | plcN_2 | Non-hemolytic phospholipase C | 3.1.4.3 | COG3511 | 2,193 | 84% |
|  | CDS_02665 | cdiA_3 | 16S rRNA endonuclease CdiA | 3.1.-.- | COG3210 | 10,692 | 83% |
|  | CDS_01719 | plcN_1 | Non-hemolytic phospholipase C | 3.1.4.3 | COG3511 | 2,079 | 81% |
| Pa-PAK | CDS_05381 | estA | Esterase EstA | 3.1.1.1 | COG3240 | 1,941 | 92% |
|  | CDS_02626 | rhsC_3 | Putative deoxyribonuclease RhsC | 3.1.-.- | COG3209 | 2,664 | 90% |
|  | CDS_01601 | rhsC_1 | Putative deoxyribonuclease RhsC | 3.1.-.- | COG3209 | 2,658 | 88% |
|  | CDS_04314 | plcN_2 | Non-hemolytic phospholipase C | 3.1.4.3 | COG3511 | 2,193 | 86% |
|  | CDS_00042 | cdiA | 16S rRNA endonuclease CdiA | 3.1.-.- | COG3210 | 10,512 | 86% |
|  | CDS_01650 | plcN_1 | Non-hemolytic phospholipase C | 3.1.4.3 | COG3511 | 2,079 | 83% |
| Pa-PAO1 | CDS_05276 | estA | Esterase EstA | 3.1.1.1 | COG3240 | 1,941 | 90% |
|  | CDS_00868 | plcN_1 | Non-hemolytic phospholipase C | 3.1.4.3 | COG3511 | 2,193 | 86% |
|  | CDS_00042 | cdiA_1 | 16S rRNA endonuclease CdiA | 3.1.-.- | COG3210 | 10,608 | 84% |
|  | CDS_03435 | plcN_2 | Non-hemolytic phospholipase C | 3.1.4.3 | COG3511 | 2,079 | 82% |
|  | CDS_02812 | hsdR | Type-1 restriction enzyme R protein | 3.1.21.3 | NA | 3,441 | 81% |
|  | CDS_02530 | cdiA_2 | 16S rRNA endonuclease CdiA | 3.1.-.- | COG3210 | 16,884 | 81% |

**Suppl. Table 2**. Candidate phage tail depolymerases found by PDP-Miner with DePP score above 75% and Pfam domains indicating depolymerase activity, across the IPCD genome collection (N = 1,294).

| IPCD isolate | Gene | contig | start | stop | frame | Pharokka annotation | Pfam domains | DePP score |
| --- | --- | --- | --- | --- | --- | --- | --- | --- |
| IPC_1377 | CDS_5089 | scaffold7.1 | 181786 | 183660 | + | tail protein | PF22432.1:PA2794-like_C | 92% |
| IPC_1419 | CDS_6000 | scaffold22.1 | 41837 | 43900 | + | tail protein | PF22432.1:PA2794-like_C | 88% |
| IPC_38 | CDS_4033 | scaffold7.1 | 99963 | 101723 | + | lytic tail protein | PF01464.25:SLT; PF01476.25:LysM | 77% |
| IPC_133 | CDS_2732 | scaffold5.1 | 323075 | 321303 | - | lytic tail protein | PF01464.25:SLT; PF01476.25:LysM | 77% |
| IPC_1078 | CDS_1576 | scaffold3.1 | 411575 | 409803 | - | lytic tail protein | PF01464.25:SLT; PF01476.25:LysM | 77% |
| IPC_135 | CDS_3183 | scaffold6.1 | 323084 | 321312 | - | lytic tail protein | PF01464.25:SLT; PF01476.25:LysM | 76% |
| IPC_37 | CDS_3048 | scaffold4.1 | 141331 | 143091 | + | lytic tail protein | PF01464.25:SLT; PF01476.25:LysM | 76% |
| IPC_1607 | CDS_2631 | scaffold5.1 | 324168 | 322396 | - | lytic tail protein | PF01464.25:SLT; PF01476.25:LysM | 76% |
| IPC_1751 | CDS_3739 | scaffold6.1 | 323078 | 321306 | - | lytic tail protein | PF01464.25:SLT; PF01476.25:LysM | 75% |
| IPC_1592 | CDS_2758 | scaffold6.1 | 323035 | 321263 | - | lytic tail protein | PF01464.25:SLT; PF01476.25:LysM | 75% |

**Suppl. Table 3.** Benchmarking PDP-Miner, PhageDPO and DepoScope predictions for all tail putative tail protein genes from 5 complete phage genomes known to produce depolymerases. PFAM domain descriptions are provided below this summary table.

| **NCBI Accession // Phage genome** | **Tail protein genes** | | | | **PFAM domains** | | **Prediction scores (%)** | | |
| --- | --- | --- | --- | --- | --- | --- | --- | --- | --- |
|  | **CDS** | **Start** | **Stop** | **Pharokka Annotation** | **Names** | **Accessions** | **PDP-Miner** | **Phage-DPO** | **Depo-Scope** |
| NC_048162.1 // Klebsiella phage GH-K3 | CDS_0034 | 26369 | 25632 | Minor tail | Prok-JAB; NLPC_P60 | PF14464.11; PF00877.24 | 38 | 0 | 0 |
|  | CDS_0035 | 27123 | 26371 | Minor tail | Phage_tail_L | PF05100.17 | 32 | 4 | 0 |
|  | CDS_0036 | 27537 | 27193 | Minor tail | Phage_min_tail | PF05939.18 | 17 | 5 | 0 |
|  | CDS_0039 | 31919 | 31263 | Major tail | Phage_tail_3 | PF08813.16 | 37 | 62 | 0 |
| NC_028987.2 // Acinetobacter phage IME200 | CDS_0037 | 25397 | 25582 | Tail | None found | None found | 7 | 5 | 0 |
|  | CDS_0040 | 26597 | 28888 | Tail | None found | None found | 96 | 93 | 100 |
| NC_013647.1 // Klebsiella phage KP32 | CDS_0037 | 23058 | 23636 | Tail | Tube | PF17212.8 | 29 | 0 | 0 |
|  | CDS_0038 | 23659 | 26034 | Tail | None found | None found | 94 | 91 | 100 |
| NC_029099.1 //  Klebsiella phage KP36 | CDS_0040 | 18580 | 19236 | Major tail | Phage_tail_3 | PF08813.16 | 37 | 51 | 0 |
|  | CDS_0043 | 22959 | 23303 | Minor tail | Phage_min_tail | PF05939.18 | 19 | 5 | 0 |
|  | CDS_0044 | 23373 | 24125 | Minor tail | Phage_tail_L | PF05100.17 | 33 | 3 | 0 |
|  | CDS_0045 | 24127 | 24864 | Minor tail | Prok-JAB; NLPC_P60 | PF14464.11; PF00877.24 | 35 | 0 | 0 |
|  | CDS_0048 | 32160 | 29509 | Tail | None found | None found | 97 | 99 | 100 |
| NC_025418.1 // Klebsiella phage  NTUH-K2044-K1-1 | CDS_0040 | 26714 | 29086 | Tail | None found | None found | 97 | 82 | 100 |
|  | | | | | | | | | |
| **PFAM domains** | **Description** | | | | | | | | |
| PF14464.11:Prok-JAB | This entry represents the JAB domain in prokaryotes. The domain is widely found in bacteria, archaea and phages. Its function is still not clear. Source: <https://www.ebi.ac.uk/interpro/entry/pfam/PF14464/> | | | | | | | | |
| PF00877.24:NLPC_60 | The NlpC/P60 family includes a number of characterised bacterial cell wall hydrolases. Members of this related family are all found in prophage regions of bacterial genomes. Source: <https://www.ebi.ac.uk/interpro/entry/InterPro/IPR011929/> | | | | | | | | |
| PF05100.17:Phage_tail_L | This entry represents Tail tip protein (L) from Bacteriophage lambda and similar proteins found in tailed bacteriophages (Caudovirales) and prophages mostly from Proteobacteria. Source: <https://www.ebi.ac.uk/interpro/entry/pfam/PF05100/> | | | | | | | | |
| PF05939.18:Phage_min_tail | This family consists of a series of phage minor tail proteins and related sequences from several bacterial species. Source: <https://www.ebi.ac.uk/interpro/entry/pfam/PF05939/> | | | | | | | | |
| PF08813.16:Phage_tail_3 | This is a family of phage tail tube proteins. A few members have an associated bacterial Ig-like domain, PF02368, at their C-terminus. Source: <https://www.ebi.ac.uk/interpro/entry/pfam/PF08813/> | | | | | | | | |
